# A Mixed Iteration Method to Determine the Linear Material Parameters in the Study of Creep Behavior of the Composites

**DOI:** 10.3390/polym13172907

**Published:** 2021-08-29

**Authors:** Mostafa Katouzian, Sorin Vlase, Maria Luminița Scutaru

**Affiliations:** 1 Department Machine Tools, Technical University of Munich, 85748 Munich, Germany; d-mec@unitbv.ro; 2 Department of Mechanical Engineering, Transilvania University of Brașov, B-dul Eroilor 20, 500036 Brașov, Romania; 3 Romanian Academy of Technical Sciences, B-dul Dacia 26, 030167 Bucharest, Romania

**Keywords:** composite, creep behavior, constitutive low, fibrous material

## Abstract

This paper presents and applies a mixed iteration method to determine the nonlinear parameters of the material used to study a composite’s creep behavior. To describe the research framework, we made a synthetic presentation of the viscoelastic behavior of composite materials by applying classical models. Further, the presented method was based on a calculation algorithm and program, which was applied on several types of materials. In a consecutive procedure of experiments and calculations, we determined the material parameters of the studied materials. The method was further applied to two composite materials in which the nonlinearity factors at different temperatures were determined.

## 1. Introduction

In viscoelastic materials, creep phenomena are currently found in engineering applications. Creep is a permanent deformation caused by a long mechanical stress. The phenomenon of creep is time dependent. A deformation rate is defined, which depends mainly on the properties of the studied material, in addition to the temperature and level of the mechanical load [1,2,3]. For materials frequently used in engineering, temperature is the main factor that influences the rate of deformation of the material [4,5]. The creep phenomenon can be extremely harmful in some cases and can lead to the destruction of an entire part or even the whole assembly. There is generally a significant increase in the rate of deformation, which occurs near the melting point, usually at strong heating, yet there are some cases in which the creep phenomenon can significantly manifest itself at room temperature. This, which is generally not necessary when designing a technical assembly, can be an undesirable phenomenon in most practical cases and can lead to significant damage.

The most suggestive example of this is polymers, which are frequently used in technical applications and can present creep phenomena at room temperature or at relatively low operating temperatures [6,7]. Because polymers are extensively used in the manufacturing of composite materials, their behavior and creep has been an intensively studied area in recent decades [8]. This is mainly due to the engineering applications in which composite materials are involved, such as their use in the automotive and civil engineering, aeronautics, and medicine, as well as many other fields of activity. Based on the previous experience of the designer, if the occurrence of the creep phenomenon of some materials from the built mechanical system is presumed, then the deformation rate must be known based on experiments or corresponding theoretical models. In this way, only a proper technical project can be obtained. To achieve this goal, laboratory experiments were performed [9]. However, at the same time, sophisticated mathematical models were developed [10,11,12,13]. These results were synthesized in [14].

Most studies have implemented Schapery’s formulation [15]. Schapery’s theory states that the viscoelastic behavior of a material is defined by the parameters $g_{o}$, $g_{1}$, $g_{2}$, and $a_{\sigma }$ that describe the deviation in relation to the linear viscoelastic response.

Using this model, we proposed a new methodology for estimation of the nonlinear viscoelastic parameters, $g_{o}$, $g_{1}$, and $a_{\sigma }$ [16]. The validity of the results obtained was verified with the help of creep-recovery experiments. 

In general, in the analysis of fiber-reinforced composites, it is assumed that the fibers are composed of an elastic and linear anisotropic material, and the matrix is a material with a nonlinear viscoelastic behavior. In the characterization of the viscoelastic materials, it is essential to differentiate between linear and nonlinear material behavior. It is therefore important to recognize the conditions that must be satisfied if linearity is to be assumed. For composite materials, both criteria (see Section 2) define the linear behavior and must be verified to be considered a linear behavior of the matrix material. In practice, there are important composite materials that meet the criterion considered for linearity, and yet these materials are very nonlinear [10]. Perhaps a word of caution should be added regarding when to attribute linear viscoelastic behavior to a matrix. Even at modest composite loadings, portions of the matrix may be sufficiently highly loaded so that the composite response may be nonlinear. This can possibly cause errors in theoretical predictions.

This paper presents a mixed iteration method used to determine the linear material parameters in the creep models for composites. It was necessary to obtain experimental results for the studied materials. These experiments allowed us to determine the nonlinearity factors for two composite materials. 

## 2. Viscoelastic Models 

Here, we describe two criteria used to determine a linear response of a material.

(i) Homogeneity

The property states that a proportional change in input history causes the same proportional change in response. In other words, if the stress (input) is doubled, the strain response (output) doubles. This property is express with the relation:

(1) ε(kσ(t))=kε(σ(t))

(ii) Superposition

This property on the other hand, which is not restricted to proportional changes, states that the response due to sum of two or more inputs is identical to the sum of responses due to each input applied separately, i.e.,

(2) $$\varepsilon \left(\sigma_{I}(t)+\sigma_{II}(t)+\ldots \right)=\varepsilon \left(\sigma_{I}(t)\right)+\varepsilon \left(\sigma_{II}(t)\right)+\ldots$$

If a material meets condition (ii), it automatically satisfies the condition of proportionality (i). However, the reciprocal is not valid. The hypothesis made by many researchers in their work that the property of homogeneity is sufficient for an answer, cannot be justified and the answer of the material can be strongly nonlinear. In conclusion, it is essential to apply the overlap test when studying the behavior of a linear viscoelastic material. As mentioned earlier, Boltzmann’s overlapping principle is a consequence of the linear behavior of the material, which applies only in the linear domain. As stated earlier, the Boltzmann’s superposition principle is a consequence of linear behavior of the material, which is only applicable in the linear range.

There are classical rheological models known and applied in practice for the study of creep response of a viscoelastic material. These have been developed over time by many researchers [6,7]. One new and useful model is based on the method of nonlinearity factors. This method was developed by Schapery [17,18] and has been used successfully by several investigators [19,20,21]. This paper is a contribution to the development of this method. The nonlinear viscoelastic constitutive equation for uniaxial creep type loading is as follows [17]:

(3) $$\varepsilon (t)=g_{o}D_{o}\sigma +g_{1}\int_{-\infty }^{t}\Delta D(\varphi -\varphi^{\prime })\frac{d}{d\tau }\left(g_{2}\sigma \right)d\tau$$

where $D_{o}=D\left(0\right)$ and ΔD(φ) are the instantaneous and transient components of the linear viscoelastic creep compliance, respectively. Moreover, φ and $\varphi^{\prime }$ are reduced time parameters and are defined as:

(4) $$\varphi =\int_{0}^{t}\frac{dt^{\prime }}{a_{\sigma }}\;;\;\varphi^{\prime }=\varphi \left(\tau \right)=\int_{0}^{\tau }\frac{dt^{\prime }}{a_{\sigma }}$$

The nonlinearity factors $g_{o}$, $g_{1}$, and $g_{2}$, as well as the time shift factor $a_{\sigma }$, are stress-dependent material properties. It is readily seen that the familiar Boltzmann’s superposition principle for linear viscoelastic behavior is recovered by setting $g_{o}=g_{1}=g_{2}=a_{\sigma }=1$. Furthermore, Leadermann’s modified superposition principle (MSP), introduced earlier [7], can be obtained by substituting $g_{o}=g_{1}=a_{\sigma }=1$ in Equation (3), yielding:

(5) $$\varepsilon (t)=D_{o}\sigma +\int_{0}^{t}\Delta D(t-\tau )\frac{d}{d\tau }(g_{2}\sigma )d\tau$$

The nonlinearity factors in Equation (3) have certain thermodynamic significance. According to Schapery [22], changes in $g_{o}$, $g_{1}$, and $g_{2}$ arise from the third and higher order dependence of Gibbs free energy on the applied stress, while changes in $a_{\sigma }$ reflect a similar dependence of both entropy production and free energy. 

For the stress history of a creep experiment $\sigma =\sigma_{a}H(t)$, where $\sigma_{a}$ = constant applied creep stress and H(t) = Heaviside’s unit step function, a constant test temperature (*T*′) can be written as:

(6) $$\varepsilon (t,T^{\prime },\sigma_{a})=\sigma_{a}\left[g_{o}D_{o}+g_{1}g_{2}\Delta D(t/a_{\sigma })\right]$$

It has been shown that excellent modeling of the isothermal creep response of polymers is achieved through the assumption $a_{\sigma }=1$ [23,24].

The time dependence of the linear creep compliance is approximated in the current study by a Prony-Dirichlet series:

(7) $$D(t,T^{\prime })=D_{o}+\sum_{j=1}^{m}D_{j}\left(1-e^{-t/\tau_{j}}\right)$$

where $\tau_{1},\tau_{2},\ldots ,\tau_{m}$ are discrete retardation times and $D_{0}(T^{\prime }),D_{1}(T^{\prime }),\ldots ,D_{m}(T^{\prime })$ represent constant material parameters at the given temperature ($T^{\prime }$). Using the notation $g_{t}=g_{1}g_{2}$ [24], it follows from Equations (6) and (7) that for the nonlinear creep compliance:

(8) $$D_{n}(t,T^{\prime },\sigma_{a})=\frac{\varepsilon }{\sigma_{a}}=g_{o}D_{o}+g_{t}\sum_{j=1}^{m}D_{j}\left(1-e^{-t/\tau_{j}}\right)$$

Note that the factor $g_{o}$ describes the nonlinearity of the instantaneous response, while $g_{t}$ characterizes the nonlinearity of the transient creep response. Based on creep tests performed at the constant temperature *T*′ and under different stress levels $\sigma_{o},\sigma_{1},\ldots ,\sigma_{l}$ the *m* + 1 linear material parameters $D_{0}(T^{\prime }),D_{1}(T^{\prime }),\ldots ,D_{m}(T^{\prime })$ and the l + 1 nonlinearity factors $g_{o}(\sigma_{i},T^{\prime })$ and $g_{t}(\sigma_{i},T^{\prime })$ of (8) can be evaluated. In the present paper, a numerical procedure based on the least squares techniques [25] is used to compute the above parameters and nonlinearity factors. The stress dependence of the nonlinearity factors is determined by performing creep tests at various temperatures $T^{\prime }=T_{o},T_{1},\ldots ,T_{n}$. The factors $g_{o}(\sigma ,T)$ and $g_{t}(\sigma ,T)$ are then approximated by suitable mathematical functions.

For a complete description of materials exhibiting temperature-dependent nonlinear viscoelastic behavior, the temperature dependence of the linear material parameters $D_{0}(T),D_{1}(T),\ldots ,D_{m}(T)$ should also be determined.

**Figure 1:**
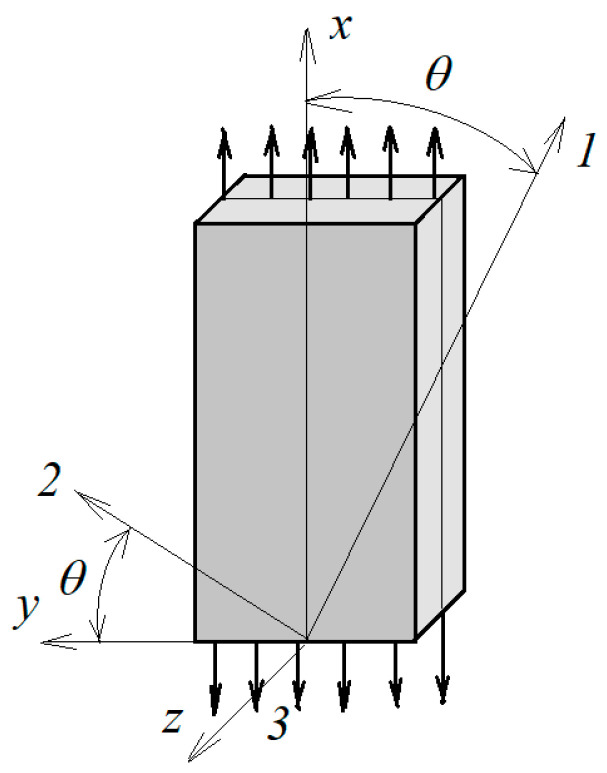
Anisotropic tensile specimen.

For a single lamina having an orthotropic material property, the compliance matrix [D] contains four principal independent compliances $D_{11},D_{12},D_{22},D_{66},(D_{21}=D_{12})$. For continuous fiber-reinforced composites (see Figure 1), the compliances $D_{11}$ and $D_{12}$ exhibit negligible viscoelastic behavior [26,27]; hence, these terms can be taken as time-independent. If environmental and aging effects are excluded, [D] has the following form:

(9) $$\left[D\right]=\left[\begin{array}{ccc} D_{11}(t,T,\sigma ) & D_{12}(t,T,\sigma ) & 0\\ D_{21}(t,T,\sigma ) & D_{22}(t,T,\sigma ) & 0\\ 0 & 0 & D_{66}(t,T,\sigma ) \end{array}\right]$$

To evaluate $D_{66}$, which is used to measure time-dependent shear modulus $G_{12},{\left[\pm 45\right]}_{2s}$ laminates are tested under tensile creep loading condition similar to that of 90-degree specimens. Using the transformation presented in [28] for $\theta =45^{o}$(the angle between global and local coordinates (see Figure 1)), it can be shown that:

(10) $$G_{12}=\frac{\sigma_{x}}{2\left(\varepsilon_{x}-\varepsilon_{y}\right)}$$

where *x* and *y* are the longitudinal and transverse directions. Moreover, the shear stress is found to be $\tau_{12}=\sigma_{x}/2$, with $\sigma_{x}$ being the constant applied stress in the creep test. The above laminate configuration is therefore used to evaluate the in-plane distortion defined as $\gamma_{12}=\varepsilon_{x}-\varepsilon_{y}$.

The constitutive equation (in uniaxial stress and isothermal conditions) is written as such [1]:

(11) $$\varepsilon (t)=g_{o}D_{o}\sigma (t)+g_{1}\int_{0}^{t}\Delta D(\varphi -\varphi^{\prime })\frac{d}{d\tau }\left[g_{2}\sigma (t)\right]d\tau$$

where $D_{o}$ is the initial (time-dependent) portion of the compliance and ΔD(φ) is the transient value of the creep compliance. The reduced times is as follows:

(12) $$\varphi =\varphi (t)=\int_{0}^{t}\frac{dt^{\prime }}{a_{\sigma }\left[\sigma (t^{\prime })\right]}\;,\;\left(a_{\sigma }>0\right)$$

(13) $$\varphi \prime =\varphi (\tau )=\int_{0}^{\tau }\frac{dt^{\prime }}{a_{\sigma }\left[\sigma (t^{\prime })\right]}\;,\;\left(a_{\sigma }>0\right)$$

Although $a_{\sigma }$ is written here as to depend on the applied stress, it may very well be a function of environmental condition, i.e., temperature and moisture. 

**Figure 2:**
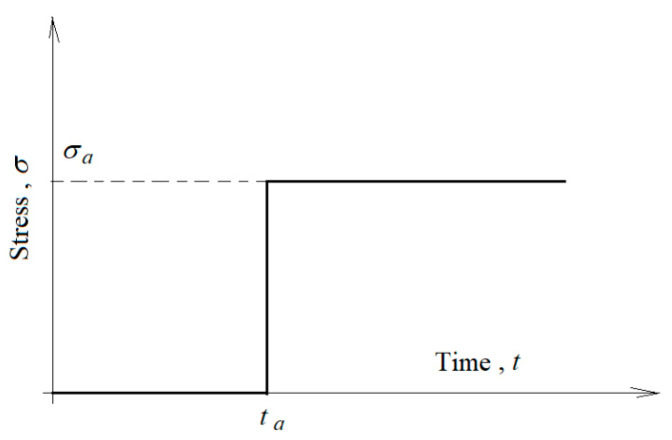
Stress versus time for a single-step input.

Suppose that a body is under the stepwise stress history, as shown in Figure 2, or

(14) $$\sigma (t)=\sigma_{a}H(\tau -t_{a})$$

where:

(15) $$H(\tau -t_{a})=\{\begin{array}{c} 0\;for\;\tau <t_{a}\\ 1\;for\;\tau >t_{a} \end{array}$$

where H(…) is the unit step function with its derivative known as the Dirac delta function δ(…). In an attempt to simplify the terms in Equation (11), let us first consider the second term in the integral, which by using the above definition can be written as:

(16) $$\frac{d}{d\tau }\left[g_{2}\sigma (t)\right]=\frac{d}{d\tau }\left[g_{2}\sigma_{a}H(\tau -t_{a})\right]$$

Since the parameter $a_{\sigma }$ in the reduced times φ and $\varphi^{\prime }$ is also required to be constant, it can be taken out of the integral when evaluating these integrals:

(17) $$\varphi =\int_{0}^{t}\frac{dt^{\prime }}{a_{\sigma }}={\frac{t^{\prime }}{a_{\sigma }}|}_{0}^{t}\;,\;$$

and:

(18) $$\varphi^{\prime }=\int_{0}^{t}\frac{dt^{\prime }}{a_{\sigma }}={\frac{t^{\prime }}{a_{\sigma }}|}_{0}^{\tau }=\frac{\tau }{a_{\sigma }}\;\;$$

In these general equations, *t* represents all times (i.e., the total time history of composite), whereas $t_{a}$ is the time at which stress $\sigma_{a}$ (constant for creep loading) is applied [29,30,31,32]. Substitution of the above equation into Equation (11) yields:

(19) $$\varepsilon (t)=g_{o}D_{o}\sigma_{a}(t)H(\tau -t_{a})+g_{1}g_{2}\sigma_{a}\int_{0}^{t}\Delta D(\frac{t}{a_{\sigma }}-\frac{\tau }{a_{\sigma }})\delta (\tau -t_{a})d\tau ,\;$$

This equation is valid for times greater than that which corresponds to the application of the constant stress $\sigma_{a}$. It can be simplified to:

(20) $$\varepsilon (t)=\left[g_{o}D_{o}+g_{1}g_{2}\Delta D(\frac{t-\tau }{a_{\sigma }})\right]\sigma_{a},$$

or, alternatively, to

(21) $$\varepsilon (t)=D_{n}\sigma_{a}$$

with the nonlinear creep compliance defined as:

(22) $$D_{n}=\frac{\varepsilon (t)}{\sigma_{a}}=g_{o}D_{o}+g_{1}g_{2}\Delta D(\frac{t-\tau }{a_{\sigma }})$$

The nonlinear creep compliance is composed of a time-independent portion and a transient part, with the latter being a function of the reduced time. The above expression can equivalently be written in the following form:

(23) $$D_{n}=\frac{\varepsilon (t)}{\sigma_{a}}=g_{o}D_{o}+g_{t}\Delta D(\frac{t-\tau }{a_{\sigma }})$$

with $g_{o}$ and $g_{t}=g_{1}g_{2}$ [24] referred to as time-independent and transient nonlinearity factors, respectively. For the case when stress $\sigma_{a}$ is applied at τ=0, the nonlinear creep compliance is simplified to:

(24) $$D_{n}=\frac{\varepsilon (t)}{\sigma_{a}}=g_{o}D_{o}+g_{t}\Delta D(\frac{t}{a_{\sigma }})$$

## 3. Determination of the Linear Material Parameters Using “Mixed” Iterations

**Figure 3:**
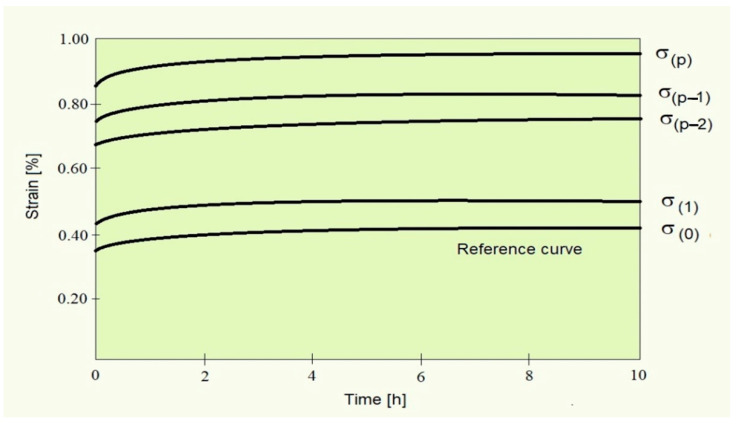
Strain versus time in a creep typical experiment.

We [24] evaluated linear viscoelastic parameters directly from experimental data obtained from experiments conducted in linear and nonlinear ranges. The nonlinearity factors were computed separately for each nonlinear test. Here, we illustrate the applicability of the above method using an example on a typical creep experiment for which the following diagram is shown (Figure 3).

The creep strain for a material in the nonlinear loading range under isothermal environmental condition is written as:

(25) $$\varepsilon (t,\sigma )=\sigma_{a}\left[g_{o}D_{o}+g_{t}\sum_{j=1}^{m}D_{j}\left(1-e^{-t/\tau_{j}}\right)\right]$$

where $\sigma_{a}$ is the constant applied creep stress. The linear viscoelastic loading case is recovered by setting the nonlinearity factors $g_{o}=g_{t}=1$, which corresponds to the lowest curve shown in the above diagram. The first requirement when applying the above method is that the nonlinearity factors are known not only for the linear test for which $g_{o}=g_{t}=1$ [26] but also for the remaining “*p*” tests conducted in the nonlinear range.

For each of the experimentally determined creep curves with applied uniaxial stress ($\sigma_{ak}$), the following constitutive relation can be written:

(26) $$\varepsilon_{ki}=\sigma_{ak}\left[g_{ko}D_{o}+g_{kt}\sum_{j=1}^{m}D_{j}\left(1-e^{-t/\tau_{j}}\right)\right]$$

The index “*k*” refers to the applied stress level, while “*i*” denotes the time at which the measurement is made. According to the above diagram, the indices in Equation (36) cover the following ranges:

i=1,2,……,n (the same for all tests) and represent the number of the time measurement;j=1,2,……,m (linear material parameters);
k=0,1,2,……,p (*k* = 0 refers to the linear test) the number of different axial stressors applied during the experiment.
The time $t=t_{i}$ corresponds to the creep strain values $\varepsilon_{0}^{*}(t),\varepsilon_{1}^{*}(t),\ldots ,\varepsilon_{p-1}^{*}(t),\varepsilon_{p}^{*}(t)$, which are measured after the same time interval as the beginning of the experiment.

The above measured values, however, may deviate from those computed by theoretical values for a single creep test. As such, a total *n*(*p* + 1) of such differences should exist. This would correspond to the entire set of the creep tests, as well as to the (different) times at which the measurements are made. These errors must be kept at a minimum by using the least squares method, which follows that

(27) $$\{\begin{array}{c} I_{oi}=g_{00}D_{0}+g_{0t}\sum_{j=1}^{m}D_{j}\left(1-e^{-t_{i}/\tau_{j}}\right)-\frac{\varepsilon_{0i}^{*}}{\sigma (0)}\\ I_{1i}=g_{10}D_{0}+g_{1t}\sum_{j=1}^{m}D_{j}\left(1-e^{-t_{i}/\tau_{j}}\right)-\frac{\varepsilon_{1i}^{*}}{\sigma (1)}\\ \vdots \\ I_{pi}=g_{p0}D_{0}+g_{pt}\sum_{j=1}^{m}D_{j}\left(1-e^{-t_{i}/\tau_{j}}\right)-\frac{\varepsilon_{pi}^{*}}{\sigma (p)} \end{array}$$

or written equivalently as:

(28) $$I_{ki}=g_{k0}D_{0}+g_{kt}\sum_{j=1}^{m}D_{j}\left(1-e^{-t_{i}/\tau_{j}}\right)-\frac{\varepsilon_{ki}^{*}}{\sigma (k)}\;;k=0,1,2,\ldots ,p$$

Let $\tilde{I}$ represent the sum of the above-mentioned *n*(*p* + 1) squared errors, where

(29) $$\tilde{I}={\sum_{k=0}^{p}\sum_{i=0}^{n}\left[g_{k0}D_{0}+g_{kt}\sum_{j=1}^{m}D_{j}\left(1-e^{-t_{i}/\tau_{j}}\right)-\frac{\varepsilon_{ki}^{*}}{\sigma (k)}\right]}^{2}.$$

In order for this sum to be a minimum, the following conditions must be satisfied:

(30) $$\frac{\partial \tilde{I}}{\partial D_{0}}=0$$

and

(31) $$\frac{\partial \tilde{I}}{\partial D_{l}}=0\;,\;l=1,2,\ldots \ldots ,m.$$

This, in turn, means that:

(32) $$\sum_{k=0}^{p}\sum_{i=0}^{n}\left[g_{k0}D_{0}+g_{kt}\sum_{j=1}^{m}D_{j}\left(1-e^{-t_{i}/\tau_{j}}\right)-\frac{\varepsilon_{ki}^{*}}{\sigma (k)}\right]g_{k0}=0\;\sum_{k=0}^{p}\sum_{i=0}^{n}\left[g_{k0}D_{0}+g_{kt}\sum_{j=1}^{m}D_{j}\left(1-e^{-t_{i}/\tau_{j}}\right)-\frac{\varepsilon_{ki}^{*}}{\sigma (k)}\right]g_{kl}\left(1-e^{-t_{i}/\tau_{j}}\right)=0,\;l=1,2,\ldots \ldots ,m$$

The above *m* + 1 equations contain the *m* + 1 linear material parameters $D_{0},D_{1},\ldots ,D_{m}$ and after a series of simple mathematical operations and groupings of terms, one can write, briefly:

(33) $$\left\lfloor \left(g^{T}\cdot g\right)*\left(A^{T}\cdot A\right)\right\rfloor \cdot D=\left\lfloor g^{T}*\left(A^{T}\cdot D^{*}\right)\right\rfloor \cdot I$$

where:

⋅—represents conventional matrix multiplication.
*—represents “term by term” multiplication (as in matrix addition).
*T*—indicates a transposed matrix.

The matrices in Equation (33) can be written as:

(34) $$A=\left[\begin{array}{ccccc} 1 & \left(1-e^{-t_{1}/\tau_{1}}\right) & \left(1-e^{-t_{1}/\tau_{2}}\right) & \ldots & \left(1-e^{-t_{1}/\tau_{m}}\right)\\ 1 & \left(1-e^{-t_{2}/\tau_{1}}\right) & \left(1-e^{-t_{2}/\tau_{2}}\right) & \ldots & \left(1-e^{-t_{2}/\tau_{m}}\right)\\ \vdots & \vdots & & \ldots & \\ \vdots & \vdots & & \ldots & \\ 1 & \left(1-e^{-t_{n}/\tau_{1}}\right) & \left(1-e^{-t_{n}/\tau_{2}}\right) & \ldots & \left(1-e^{-t_{n}/\tau_{m}}\right) \end{array}\right]$$

and:

(35) $$g=\left[\begin{array}{ccccc} g_{00} & g_{0t} & g_{0t} & \ldots & g_{0t}\\ g_{10} & g_{1t} & g_{1t} & \ldots & g_{1t}\\ \vdots & \vdots & & \ldots & \\ \vdots & \vdots & & \ldots & \\ g_{p0} & g_{pt} & g_{pt} & \ldots & g_{pt} \end{array}\right]$$

with:

(36) $$g_{00}=g_{0t}=1;\;D=\left\{\begin{array}{c} D_{0}\\ D_{1}\\ \vdots \\ \vdots \\ D_{m} \end{array}\right\};\;\tilde{I}=\left\{\begin{array}{c} 1\\ 1\\ \vdots \\ \vdots \\ 1 \end{array}\right\}$$

and finally:

(37) $$D^{*}=\left[\begin{array}{ccccc} \frac{\varepsilon_{01}^{*}}{\sigma (0)} & \frac{\varepsilon_{11}^{*}}{\sigma (1)} & \frac{\varepsilon_{12}^{*}}{\sigma (2)} & \ldots & \frac{\varepsilon_{p1}^{*}}{\sigma (p)}\\ \frac{\varepsilon_{02}^{*}}{\sigma (0)} & \frac{\varepsilon_{12}^{*}}{\sigma (1)} & \frac{\varepsilon_{22}^{*}}{\sigma (2)} & \ldots & \frac{\varepsilon_{p2}^{*}}{\sigma (p)}\\ \vdots & \vdots & & \ldots & \\ \vdots & \vdots & & \ldots & \\ \frac{\varepsilon_{0n}^{*}}{\sigma (0)} & \frac{\varepsilon_{1n}^{*}}{\sigma (1)} & \frac{\varepsilon_{2n}^{*}}{\sigma (2)} & \ldots & \frac{\varepsilon_{pn}^{*}}{\sigma (p)} \end{array}\right]$$

In this way, it is easy to obtain the unknown vector *D*, which then allows for the calculation of nonlinear factors. It should be mentioned that the computational accuracy of the above linear material parameters is closely related to the accurate estimation of the nonlinearity factors $g_{i0}$ and $g_{it}$. It follows that in the first step of the computation, for every single test in the nonlinear range, a rough estimation of these factors is also made. The linear material parameters are then determined using the estimated values of the nonlinearity factors. 

In the next step, these factors are recalculated for every single test using the known approximated values of the linear parameters. With these nonlinearity factors, the linear material parameters computed earlier are corrected. This so-called “mixed iteration” procedure is repeated until no significant variation of the linear parameters and/or nonlinearity factors is observed. 

Using the above procedure, we can demonstrate how nonlinearity factors are determined. As previously mentioned, the test at the lowest stress level is considered as linear. This means:

(38) $$g_{00}=g_{0t}=1$$

The linear creep compliance can also be written as:

(39) $$D_{l}(t)=D_{o}+\sum_{j=1}^{m}D_{j}\left(1-e^{-t/\tau_{j}}\right)$$

As such, it follows that:

(40) $$D_{l}(0)=D_{0}$$

(41) $$D_{l}(\infty )=D_{0}+\sum_{j=1}^{m}D_{j}$$

For the nonlinear test “*i*”, it yields:

(42) $$D_{ni}(t)=g_{i0}D_{o}+g_{it}\sum_{j=1}^{m}D_{j}\left(1-e^{-t/\tau_{j}}\right)$$

and

(43) $$D_{ni}(0)=g_{i0}D_{o}$$

(44) $$D_{ni}(\infty )=g_{i0}D_{o}+g_{it}\sum_{j=1}^{m}D_{j}$$

From these equations and those in the linear range, the nonlinearity factors $g_{i0}$ and $g_{it}$ can be written as:

(45) $$g_{i0}=\frac{D_{ni}(0)}{D_{0}}=\frac{D_{ni}(0)}{D_{l}(0)}$$

(46) $$g_{it}=\frac{D_{ni}(\infty )-g_{i0}D_{0}}{\sum_{j=1}^{m}D_{j}}$$

where:

(47) $$\sum_{j=1}^{m}D_{j}=D_{l}(\infty )-D_{0}=D_{l}(\infty )-D_{l}(0)$$

thus:

(48) $$g_{it}=D_{j}$$

(49) $$g_{it}=\frac{D_{ni}(\infty )-g_{i0}D_{0}}{D_{l}(\infty )-D_{l}(0)},$$

where Equation (43) has been used.

In order to obtain a first (rough) approximation for the nonlinearity factors $g_{io}$ and $g_{it}$, it is sufficient to substitute for t=∞ with the longest experimental times. The mixed iteration procedure can then be carried out as explained above.

## 4. Experiments and Results

To obtain experimental data that allows one to estimate coefficients $g_{o}$ and $g_{t}$, it is necessary to perform tests on material specimens at different load levels and temperatures, all of which produce the creep of the respective material. The number of tests must be large enough to be statistically processed. The specimens are tested isothermally at room temperature and at higher temperatures. The tests performed at room temperature are performed at 23 °C with ambient humidity. During the experiment, the environmental conditions (humidity and temperature) are kept constant. Thus, the relative humidity ranges around ±5% RH. The samples are tested in a laboratory for a period of 10 h during which measurements are made. Laboratory experiments can be successfully used to make reasonable predictions of mechanical responses over long periods of time, such as years or even decades. In this way, the results obtained in the laboratory, in accessible time intervals, can help to predict, for long periods, the behavior of the materials.

**Figure 4:**
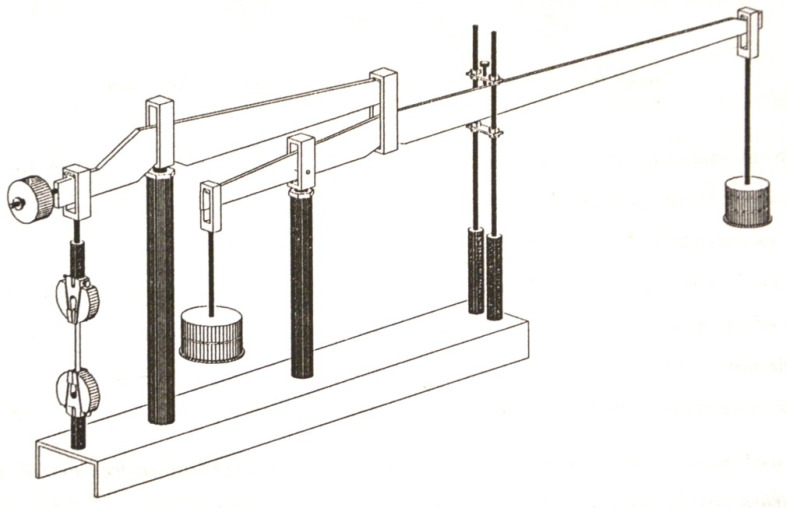
Experimental testing device [33].

Figure 4 shows the experimental testing device with two levers [33]. The experiments is performed in order to determine the behavior of the composite. The test specimens are subjected to different stress and different temperatures.

The experimental setup for the creep experiments consists of 15 test installations, which allow tensile tests. All tests can be performed simultaneously. Each installation has a maximum working load capacity from 6 kN to 30 kN. The loading level on each of the samples is determined electronically. Some of the test facilities are equipped to test at temperatures other than room temperature. Cylindrical heating chambers are attached for testing at wide temperatures. A heated, thermostatically controlled oil is pumped through the copper coil placed on the inner surface of the cylinder, along its entire length. The circulation of the heated air inside the cylinder is also ensured. The temperature of the test piece is measured using a thermocouple applied to the surface of the sample. The temperature inside the room was maintained in the range of ±1 °C. 

The materials for which the experiments are performed are two types of commercially available composites. The first material is a Fibredux 6376 epoxy resin reinforced with T800 carbon fiber (Toray Industries Europe GmbH, Germany). The epoxy resin has a glass transition temperature about 180 °C. The thermoplastic used is an APC-2 material (ICI), a semi crystalline PEEK matrix with a glass transition temperature of about 145 °C. This material is reinforced with IM6 carbon fibers (Hercules Inc.). The specimens have a nominal length of 150 mm, a width of 10 mm, and a thickness of 1 mm. The chamber has a relative humidity of about 35%.

**Figure 5:**
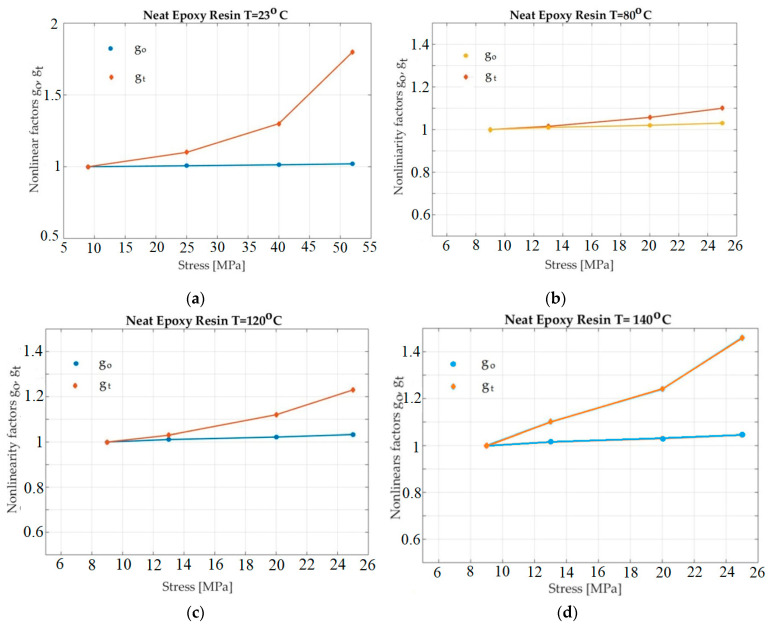
Plot of *g_o_* and *g_t_* for epoxy resin as a function of stress and temperature. (**a**)—T = 23 °C; (**b**)—T = 80 °C; (**c**)—T = 120 °C; (**d**)—T = 140 °C.

Figure 5 shows the obtained values, using the experimental data for $g_{o}$ and $g_{t}$, especially considering Neat Epoxy Resin specimens at 23 °C, 80 °C, 120 °C, and 140 °C.

The initial compliance value, *D_o_*, implies a tensile modulus *E*_22_ = 1/*D_o_* of 8.44 GPa, which is somewhat greater than the average quasistatic value of 8.04 GPa. Considering the technique by which h is measured in a static test, the reason for this discrepancy becomes clear. The creep compliance value *D_o_* is the instantaneous compliance or the reciprocal of the instantaneous elastic modulus. In a static test, the loads are not instantaneously applied, but instead the specimen is loaded in a displacement-controlled mode at a constant rate of 1–2 mm/min of a universal test machine. Stress relaxation, which occurs under such loading condition, could result in an apparent lower value for the modulus. In a creep test, however, loads are applied quicker and normally reach the applied creep stress in less than 20 s. With this in mind, the value of *E*_22_ is in accordance with the quasi-static value.

**Figure 6:**
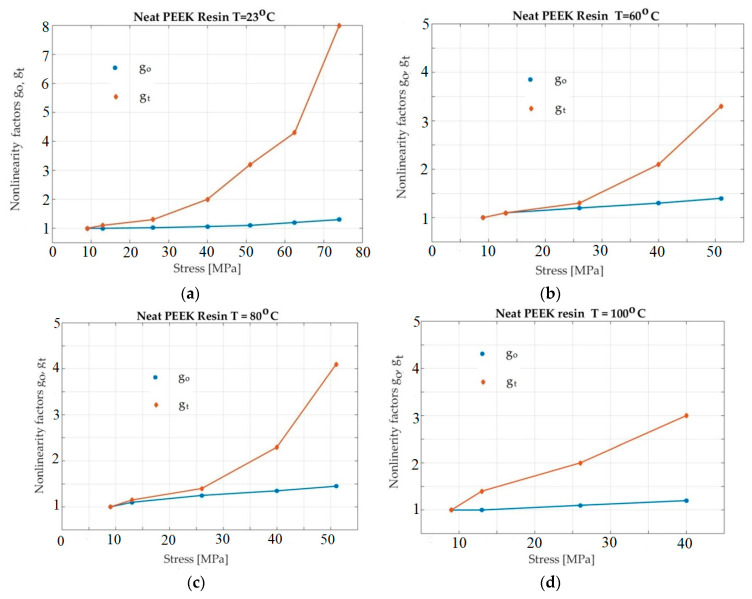
Plot of *g_o_* and *g_t_* for the neat PEEK as a function of stress and temperature: (**a**)—T = 23 °C; (**b**)—T = 80 °C; (**c**)—T = 120 °C; (**d**)—T = 140 °C.

Figure 6 shows the neat PEEK specimens for 23 °C, 60 °C, 80 °C, and 100 °C. In these experiments, it is better to observe the nonlinearity of the material behavior.

Interestingly, the creep testing procedure may be a possible source of error when generating data. Determining the point of zero strain in experimental creep testing is often connected with considerable uncertainty, which stems from the loading procedure in a creep test. This could cause a vertical shift in the entire creep curve so that higher or lower values of strain, which may differ from the actual strain in the tested specimen, could result. This could explain some of the results presented in this paper.

**Figure 7:**
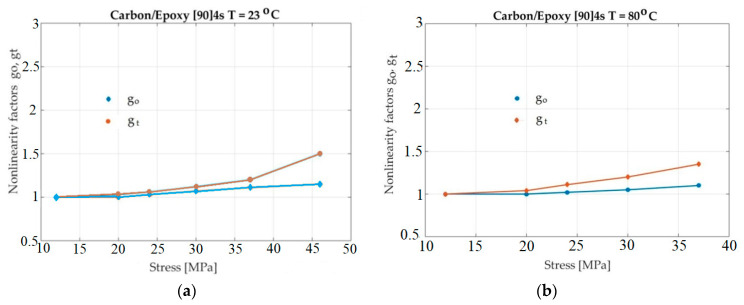

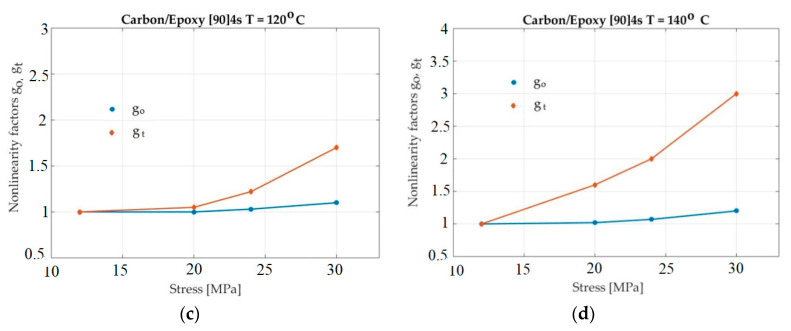
Plot of *g_o_* and *g_t_* for Carbon/Epoxy resin as a function of stress and temperature: (**a**)—T = 23 °C; (**b**)—T = 80 °C; (**c**)—T = 120 °C; (**d**)—T = 140 °C.

Additional experiments were made on a composite material made by epoxy resin reinforced with carbon fibers. The results for the nonlinearity factors, determined using the results of the experiment, are presented in Figure 7.

**Figure 8:**
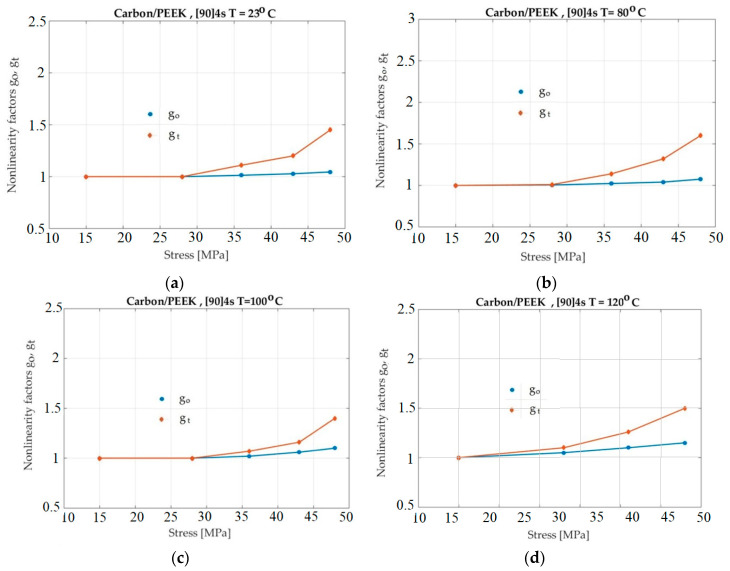
Plot of *g_o_* and *g_t_* for Carbon/PEEK as a function of stress and temperature: (**a**)—T = 23 °C; (**b**)—T = 80 °C; (**c**)—T = 120 °C; (**d**)—T = 140 °C.

Figure 8 presents the nonlinearity factors for a Carbon/PEEK specimen. A lower variation of the $g_{t}$ factor can be observed when compared to the previous case, expressing a less pronounced nonlinear behavior. The glass transition temperature of PEEK resin is about 145 °C. In these experiments, the temperature has practically no influence on situations where experimental measurements are made.

Considering Figure 6, Figure 7 and Figure 8, it seems that *g_1_* did not increase when the temperature increased. However, an objective representation of the graphs of *g_1_* for all situations show a correct situation. The four graphs for the four temperaturess (in Figure 5, Figure 6, Figure 7 and Figure 8) have different scales on the abscissa. For this reason, the four graphs cannot simply be overlapped (mentally). Before overlapping, they must be brought to a common scale on the abscissa. In this case, the dependence of *g*_1_ on temperature can be seen to be normal and natural.

In this paper, we did not intend to create a model that determined the influence of temperature on *g_t_* and *g_o_* factors for the examined cases. These cases alone are not enough to solve such a problem. A systematic study, conducted with greater temperature values, should have the ability to solve this problem.

## 5. Discussion

When analyzing the graphs presented above, several conclusions can be obtained that may prove useful for designers. First, the proposed method was found to be simple and able to solve linear systems of equations. Because this method involves laboratory measurements, the accuracy of the results depends on the number of measurements performed. Generally, when studying flow phenomena, numerous experimental determinations can be made to elucidate the behavior of the studied materials under different conditions. Here, useful values for the proposed method were easily obtained. 

Once the method is developed, useful values can be obtained. For example, for the studied materials, the following conclusions can be drawn. Because the first material tested was a pure resin specimen, the influence of significant factors (stress and temperature) on nonlinearity factors, $g_{o}$ and $g_{t}$, for the neat epoxy samples was observed (Figure 5). Obviously, the linear viscoelastic limit shifted to lower values when the temperature increased. The experiments conducted at room temperature showed a linear response for a stress level of 20 MPa. Therefore, the instantaneous creep response of the material was less sensitive to temperature than the transient response. In the test program applied, the instantaneous creep response was found to be linear up to 20 MPa. The next material studied was a PEEK resin specimen. The influence of the two factors—stress and temperature—on the nonlinearity factors, $g_{o}$ and $g_{t}$, is illustrated in Figure 6.

The primary effect of temperature was found to be similar to that of the neat epoxy. When using room temperature, a linear response up to 25 MPa creep stress was noticed. When the temperature increased to 100 °C, this value reduced to 9 MPa. For the neat epoxy resin, the influence of temperature on the sudden response was less critical than on the transient response. Furthermore, the instantaneous response for all temperatures was about 30 MPa. In this case, we found that the nonlinearity factors (see Figure 7) showed that temperature had a greater influence on the transient response than on the instantaneous response. When comparing the nonlinearity factors of the reinforced laminates with those of the neat epoxy, we observed a 120 °C temperature on the linear viscoelastic limit, which was rather insignificant. In other worlds, between 23 °C and 120 °C indicates that the strain response is linear up to 18 MPa. For the higher temperature (140 °C), this limit reduced to 13 MPa, which was a predictable result. Moreover, the influence of temperature on the transient response was shown to be more significant for the composite material than for the neat resin.

Figure 8 shows a 90-degree specimen with a linear viscoelastic behavior limit slightly affected by temperature. It can be seen that the nonlinear behavior begun at 25 MPa. This behavior was similar to the behavior previously presented for the epoxy resin. These results represents an interesting observation: if a more or less temperature-independent linear behavior can be assumed to prevail up to a stress level of 25 MPa, the loading range of interest for $D_{22}$ can nearly be covered.

## 6. Conclusions 

In this paper, we presented an application for two different materials and our results were obtained from the ensuing measurements. This method is useful when laboratory measurements are performed, as they successfully characterize the examined materials and creep phenomenon.

The cases analyzed in this paper show that the best-fitted curves were drawn through data points to describe $g_{o}$ and $g_{t}$ with stress at each temperature. To more accurately define the trend of the curves, more data points are needed at high stress levels. 

The analysis and methods presented in this study proved to be effective tools for determining time-dependent responses to unidirectional composites under various loading conditions with reasonable accuracy. It was necessary to determine which mechanical properties of the materials were to be used in the creep behavior study of the composite materials. This was done via experimental determinations processed with the obtained data. 

This paper presents a method that capitalizes on experimental data obtained from measurements. Further, it allows one to obtain linear viscoelastic parameters using experimental data obtained from all experiments, both linear and nonlinear. The nonlinear factors are then calculated separately for each nonlinear test. Using a larger number of data in the performed calculations, it is expected that the results obtained will be more accurate, which is the advantage of the proposed method. Moreover, the calculation of these nonlinear parameters can lead to an increased calculation effort [15], as well as the use of some theoretical models, against which the mixed iteration method remains a more precise method of determining them.

## Data Availability

Not applicable.
